# Effectiveness of virtual clinical simulation in palliative care education for nursing students: Protocol of a randomized controlled trial

**DOI:** 10.1371/journal.pone.0349168

**Published:** 2026-05-28

**Authors:** Zhixiang Sun, Pingpin Wen, Lu Zhang, Xiaoli Zhang, Xinyu Yang, Chunyan Liu, Jing Fu, Meifang Yang

**Affiliations:** 1 Wound Healing Basic Research and Clinical Application Key Laboratory of LuZhou, School of Nursing, Southwest Medical University, Luzhou, China; 2 School of Nursing, Southwest Medical University, Luzhou, China; 3 Department of Otolaryngology, Head and Neck, The Affiliated Hospital of Southwest Medical University, Luzhou, China; 4 Department of Student Affairs, The Affiliated Hospital of Southwest Medical University, Luzhou, China; 5 School of Clinical Medicine, Southwest Medical University, Luzhou, China; 6 Department of Nursing, The Affiliated Hospital of Southwest Medical University, Luzhou, China; Jouf University, SAUDI ARABIA

## Abstract

**Background:**

Palliative care often involves nursing for complex patient groups at the end of life, which requires specialized knowledge and ethical considerations. Therefore, it is full of challenges when carrying out practical teaching. Nursing students, as the next generation of healthcare professionals, will assume the important responsibility of providing specialized health services related to end-of-life care (EOLC). A deficit in direct clinical practice may impede the acquisition of essential knowledge and ability in palliative care for nursing students.Virtual clinical simulation (VCS) is acknowledged as an effective educational tool in nursing education. However, its application in palliative care education remains underexplored, particularly through rigorous study designs. While VCS has been tested in areas such as acute care or clinical deterioration, there is a global scarcity of randomized controlled trials (RCTs) evaluating its effectiveness in palliative care education, and relevant research in China remains limited. In this study protocol, we hypothesize that VCS will improve nursing students’ knowledge, ability, and attitudes regarding palliative care, compared to conventional teaching alone.

**Methods:**

This study protocol outlines a prospective RCT aimed at evaluating the educational benefits of incorporating VCS into the curriculum for nursing students at a medical university in southwestern China.These students, registered for the elective *Cultural Perspectives on Death and End-of-Life Education* will be systematically invited to join this research. The study will randomly assign 96 students into two distinct groups: a control group and an intervention group, each comprising 48 students. The control group will participate solely in conventional classroom teaching, whereas the intervention group will receive both conventional classroom teaching and enhanced learning through an additional VCS system, aimed at improving their knowledge, ability and attitudes regarding palliative care. The intervention is grounded in experiential learning theory, which supports the use of simulated environments to foster reflective practice and skill acquisition. Assessments will occur at baseline and post-intervention of the VCS, with the primary metrics for evaluation encompassing variations in students’ knowledge, ability, and attitudes concerning palliative care, as well as their satisfaction with VCS pedagogy and confidence in applying their newly acquired skills and knowledge in practical medical settings. As this is a protocol paper, no outcome data are yet available.

**Discussion:**

VCS may be an effective tool to enhance nursing students’ palliative care knowledge, ability, and attitudes, and it provides scientific evidence to inform nursing students in selecting the most appropriate approach for their palliative care education.

**Trial registration:**

Chinese Clinical Trial Registry: ChiCTR2500096377, Registered on January 22, 2025.

## 1. Introduction

Cancer poses a considerable threat to global health and human life. The most recent data from the International Agency for Research on Cancer (IARC), part of the World Health Organization (WHO), indicates that in 2022, China experienced around 4.825 million new cancer cases and 2.574 million deaths attributed to cancer. These figures represent 24.2% of the global incidence and 26.4% of the global mortality associated with this disease [1]. With the rapid movement of China into an advanced stage of demographic aging, the need for palliative care within the health care system is growing. This has brought up a pressing need to enhance education and training for healthcare professionals [2,3]. Nursing students are expected to form the backbone of the future health workforce and are entrusted with the important task of providing professional end-of-life care (EOLC) [4]. By enhancing their education in palliative care, they will be able to develop a scientific perspective on death, establish positive life values, and acquire the necessary knowledge and skills to manage and adapt to death-related events, thus providing better care to patients and their families [5]. Challenges of providing practical clinical education in palliative care to nursing students are distinct. Palliative care is a specialized medical field that involves proficiency in communication abilities for interactions with patients and healthcare professionals, compliance with ethics and morality as well as the complexity of end-of-life and death situations [6]. Many students also become sad, afraid, and anxious upon initial exposure to EOLC, which demotivates them toward it [7]. Because these challenges in terms of emotional and education may hinder the development of their knowledge and professional ability in palliative care [8]. In view of the limitations of conventional educational approaches in meeting the dynamic needs of both education and practice in palliative care [9], there is a need to devise and implement some innovative educational models in this field.

With the help of computerized platforms, the integration of the latest developments in digital and virtual technologies introduces reconstructed scenarios observed in real life and simulates role plays. Virtual clinical simulation (VCS) marks one of the giant technological developments in the field of simulation. VCS creates immersive virtual environments for clinical training, featuring dynamic virtual patients, and allows students to engage in interactive simulations directly through their screens, offering students a simulated, hands-on experience within a controlled virtual setting [10]. It is a learner-centered simulation methodology designed to develop decision-making, motor control, and communication skills, placing the individual at the core of the process [11]. VCS, as an innovative teaching approach, dynamically showcases the complex stages of EOLC, moving beyond traditional constraints of time, space, and resources to achieve educational outcomes not possible with previous models. In recent years, RCTs in countries like France, Portugal, and Singapore have implemented VCS for training nursing students. These studies have demonstrated that VCS training improves students’ professional knowledge, learning satisfaction, and self-confidence, thereby enhancing their clinical judgment ability and positively impacting their clinical practice [12–14]. Integrating VCS into practical teaching substantially enhances the training ability of students, closes the gap between theoretical learning and clinical application, and amplifies their practical and comprehensive care ability. Nevertheless, in China, there exists a significant deficiency of interventional studies that concentrate on education in palliative care, particularly RCTs assessing the effectiveness of VCS in the training of nursing students.

## 2. Methods

### 2.1. Study design

**Fig 1:**
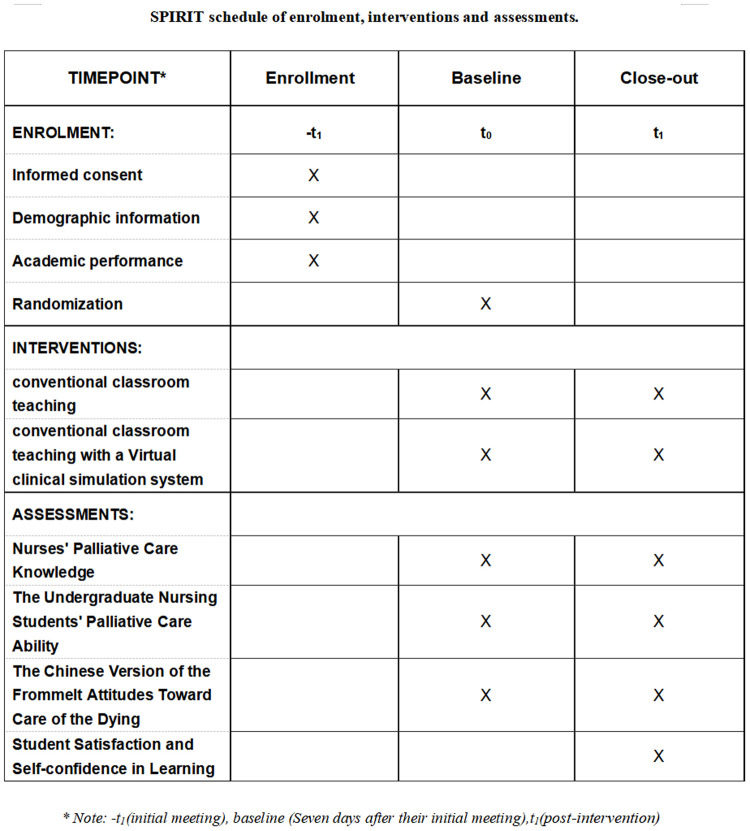
Schedule of enrollment, interventions and assessments.

**Fig 2:**
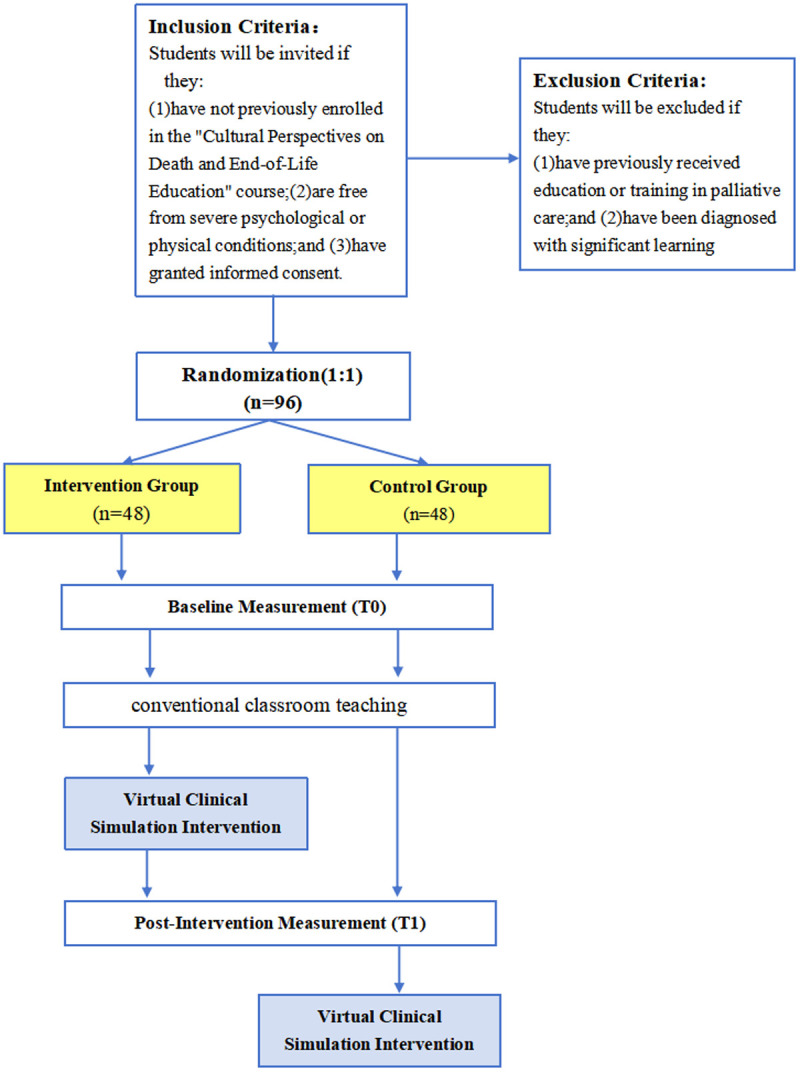
Flowchart of the study design.

This study is a prospective, single‑center randomized controlled trial (RCT) with two parallel arms, to be implemented at a medical university in Southwest China. Nursing students taking the elective course “Cultural Perspectives on Death and End-of-Life Education” at the university’s nursing department will be invited to participate. The unit of randomization is the individual student. This study protocol is developed in compliance with the Standard Protocol Items of the Recommendations for Interventional Trials (SPIRIT) guidelines (S1 File). The study will randomly assign 96 participants into two distinct groups: a control group and an intervention group, each comprising 48 students. The control group will participate solely in conventional classroom teaching, whereas the intervention group will receive both conventional classroom teaching and enhanced learning through additional VCS. The purpose of this study is to explore whether VCS, compared to conventional teaching approaches, can enhance nursing students’ knowledge, ability, and attitudes toward palliative care. The trial adheres the SPIRIT schedule Fig 1 and will be reported using the CONSORT guidelines Fig 2.

### 2.2. Ethical approval and clinical trial registration

The study has been approved by the Ethics Committee of Southwest Medical University (Approval No.:SWMUIRBTX-202501–0003). It has also been registered at the Chinese Clinical Trial Registry with the registration number ChiCTR2500096377. The Ethics Committee will review the implementation of the trial and have the authority to decide on its termination. Upon completion of the study, the research data will be uploaded to the Chinese Clinical Trials Registry. To ensure data security and confidentiality, all personal identifiers will be removed from the dataset. The unique six-digit codes chosen by participants will be used as their study identifiers, and the linkage file connecting these codes to participant names will be stored separately in a password-protected computer accessible only to the principal investigator. All data will be kept confidential and used solely for research purposes in accordance with institutional guidelines.

### 2.3. Randomization and blinding

Every student enrolled in the course will receive an invitation via email to join as research volunteers. Those who consent will participate in an initial meeting where they will sign informed consent forms. Subsequently, these volunteers will be asked to complete a detailed questionnaire that collects their sociodemographic data and academic performance, including their average course grades. This information will then be utilized to facilitate the randomization process. To preserve confidentiality, each student will choose a unique six-digit identifier. An impartial research assistant, who will not participate in the educational activities, will utilize SPSS 26.0 statistical software to allocate students evenly into either the control or intervention group with a 1:1 ratio. To ensure allocation concealment, the group assignment list will be kept separate from the research team and instructors. After randomization, group allocations will be placed in sequentially numbered, opaque, sealed envelopes, which will only be opened after participants have completed the baseline assessment. Seven days after their initial meeting，and following the completion of the randomization procedure, all students who will have volunteered for the study will be invited to meeting again. This subsequent meeting will be organized to conduct a comprehensive baseline evaluation of their knowledge, ability, and attitudes concerning palliative care.This initial assessment, referred to as the pre-intervention assessment, serves to establish a benchmark against which future improvements can be measured.During this meeting, participants will be informed of their group placement through their unique identifiers and will then be directed to the appropriate classrooms for a briefing on their respective group assignments.

Due to the nature of the intervention, blinding of participants and instructors was not possible. Participants were necessarily aware of their group allocation, and the instructors delivering the VCS could not be blinded to the intervention. However, outcome assessors and data analysts will be kept blinded to group assignment throughout the study to minimize detection bias.

### 2.4. Study participants

The study will invite nursing students who have opted for the elective course “*Cultural Perspectives on Death and End-of-Life Education*” at a medical university in Southwest China between 1 March 2026 to 30 April 2026 to participate in this study. This elective course typically enrolls approximately 100 ~ 120 students per semester. All eligible students who meet the inclusion criteria will be approached and invited to participate during the course registration period. Written informed consent will be obtained from all participants. Students will be invited if they: (1) have not previously enrolled in the “Cultural Perspectives on Death and End-of-Life Education” course; (2) are free from severe psychological or physical conditions; and (3) have granted informed consent. Students will be excluded if they: (1) have previously received education or training in palliative care; and (2) have been diagnosed with significant learning impairments. The English example of the patient consent form and the study registration protocol is provided separately in S2 File and S3 File.

### 2.5. Control Group

The control group will engage in conventional classroom teaching, which will last for 90 minutes and will be conducted by a seasoned lecturer with expertise in nursing. This teaching session will mirror the key points and utilize the same case themes as those found in the VCS system.

### 2.6. Intervention Group

Participants in the intervention group will receive the same 90 minutes of classroom teaching as the control group. Immediately following the lecture, they will additionally interact with a VCS system specifically developed for palliative care education by the research team. The VCS interaction will take place in a single, continuous session on the same day as the lecture. Participants will be able to access the VCS system repeatedly until they have completed all tasks across the three modules. The intervention session will conclude once all participants have finished their VCS tasks, allowing for varying engagement times based on individual learning pace.

### 2.7. Intervention content

**Fig 3:**
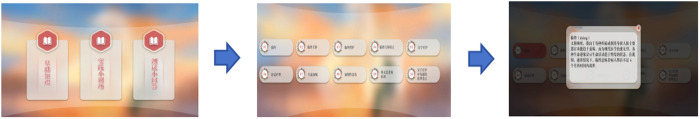
The Basic Knowledge Module Reprinted from https://virtual-emulation.zhihuishu.com/webgl/2000080450/xnykdxhospicecare202405081510/index.html?experimentId=577&courseId=2000080450&experId=5104243 under a CC BY license, with permission from Southwest Medical University, original copyright 2022.

**Fig 4:**
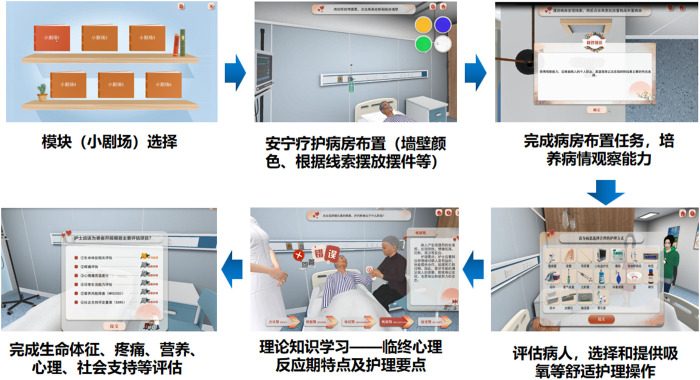
The Practice Theatre Module Reprinted from https://virtual-emulation.zhihuishu.com/webgl/2000080450/xnykdxhospicecare202405081510/index.html?experimentId=577&courseId=2000080450&experId=5104243 under a CC BY license, with permission from Southwest Medical University, original copyright 2022.

**Fig 5:**
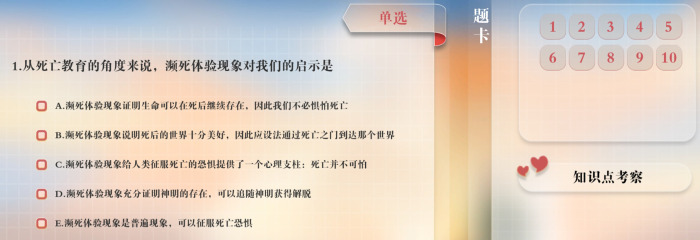
The Knowledge Testing Module Reprinted from https://virtual-emulation.zhihuishu.com/webgl/2000080450/xnykdxhospicecare202405081510/index.html?experimentId=577&courseId=2000080450&experId=5104243 under a CC BY license, with permission from Southwest Medical University, original copyright 2022.

The VCS system leverages 3D modeling technologies via Java and VRML to craft immersive environments that mimic actual hospice settings, encompassing hospital wards, medical equipment, and representations of patients, their families, and healthcare workers. Participants will assume the role of virtual nurses, engaging with non-player characters (NPCs) to adeptly navigate patient care, thereby addressing both patient and family needs. After the 90-minute conventional classroom teaching, each participant will be assigned a unique code for accessing the VCS system, which will enable research assistants to effectively track participant engagement by monitoring their login activity on the system’s backend.The VCS system is meticulously organized into three comprehensive modules: the Basic Knowledge Module, the Practice Theatre Module, and the Knowledge Testing Module. In the Basic Knowledge Module (Fig 3), participants will be introduced to basic knowledge about major concepts in palliative care. Learners will acquire a solid understanding of palliative care, its core principles, essential knowledge, psychological support for the terminally ill patient, guidelines for counselling the grieving, recognition of common clinical presentation at the end of life, and updates on EOLC practices. The Practice Theatre Module focuses on the case of an 89-year-old elderly male patient with advanced lung cancer who is admitted to a palliative care ward for hospice care (Fig 4).This module features six detailed learning scenarios: Case Introduction, Admission Care, Specialized Nursing, Spiritual Care, Social Support, and EOLC.Each scenario includes a variety of contextual educational tasks such as choices, dialogues, and reading assignments, engaging learners in decision-making processes. Learners will interact with the VCS environment by clicking on navigation icons and using menu controls on the screen, guided by an automated assistant. Additionally, the system will evaluate the “correctness” of each task choice, assign scores, and automatically log these data in the backend. These data will be used solely for providing real-time feedback to students, supporting their reflective learning; they will not be used as analytical outcome measures in this study. Such interactions will empower virtual nurses to perform assessments, physical exams, select medical tools, execute appropriate interventions, and maintain effective communication. The overarching aim is to enhance terminally ill patients’ quality of life. Furthermore, this module will seek to cultivate a compassionate attitude and holistic palliative care ability among students, thus enabling them to promptly and accurately identify and address the physiological, psychological, and emotional issues of terminally ill patients when faced with end-of-life situations. It will also promote the development of critical clinical thinking and ethical decision-making ability. The Knowledge Testing Module will present 10 randomized questions, awarding 1 point for each correct response (Fig 5).The system will generate experimental reports based on different scenarios, which encompass the correct task answers, the options selected by users, and their final scores.These reports will specify the steps or knowledge points that remain incomplete, and can be readily archived by users for future reference when conducting additional experiments. Furthermore, these reports will offer invaluable insights to facilitate students’ reflective learning processes.

### 2.8. Primary Outcomes

1. Nurses’ Palliative Care Knowledge Scale

The Palliative Care Knowledge Scale developed by Shen Yang et al. [15] is measured based on seven dimensions (34 items). Each item presents participants with three choices for responses: “True”, “False”, or “Don’t know”. Points are awarded solely for correct answers, with each accurate response earning one point, while incorrect or undecided responses (“Don’t know”) accumulate no points. The scoring range is 0 ~ 34 points, indicating a deeper and broader understanding of palliative care principles with a higher score.A Cronbach’s α coefficient of 0.947 confirms the scale’s robust reliability and validity. Dong et al administered this scale to 521 Chinese nursing students, confirming its validity in this population [16].

2 The Undergraduate Nursing Students’ Palliative Care Ability Assessment Questionnaire

The instrument developed by Zhao and her team [17] meticulously assesses three pivotal dimensions within the field of palliative care: the depth of interprofessional collaboration, the awareness of cultural and ethical issues and the proficiency in caregiving skills. It comprises 16 constructed items, each appraised using a 5-point Likert scale that spans from “no ability” at a score of 1, to “fully capable” at a score of 5. A higher score indicates better palliative care ability among nursing students, and a Cronbach’s α of 0.872 demonstrated its reliability. Ming et al administered this questionnaire to 822 undergraduate nursing students, confirming its reliability and validity [18].

3 The Chinese Version of the Frommelt Attitudes Toward Care of the Dying Scale Form B (FATCOD-B)

Nursing students and professionals often take the FATCOD-B scale, which was created by Dr. Frommelt [19] and later revised for use in China by Dr. Wang Liping [20], to assess their attitudes towards palliative care. Utilizing a 5-point Likert scale, spanning from 1 (“strongly disagree”) to 5 (“strongly agree”), this scale evaluates six dimensions and twenty-nine items. Nursing students who feel positively about EOLC tend to have higher scores.The scale is confirmed for its reliability by a Cronbach’s α coefficient of 0.796. Luo et al administered the FATCOD-B to 644 nursing students at a medical university in China, confirming its reliability and validity in this population [21].

### 2.9. Secondary Outcomes

1. Student Satisfaction and Self-confidence in Learning Scale

The National League for Nursing created this scale in 2006 [22], and Dr. Wang Ailing [23] adapted it for use in Chinese contexts in 2008. Its purpose is to measure how satisfied nursing students are with VCS education and how confident they are in their ability to apply what they have learned in future clinical practice. There are a total of thirteen items on the scale, which measure two dimensions, respectively: Satisfaction and Self-confidence in Learning. Each item uses a 5-point Likert scale, with 1 representing “strongly disagree” and 5 representing “strongly agree”. The higher the level of satisfaction and confidence nursing students have with their learning, the higher their scores will be.The satisfaction subscale has a Cronbach’s α coefficient of 0.94, while the self-confidence subscale has a coefficient of 0.72.

### 2.10. Sample Size

The determination of the necessary sample size for this investigation was conducted conducted using G*Power 3.1.9 software [24], The analysis was conducted using the t-test family, specifically for comparing the means of two independent groups. A two-tailed test was employed with an alpha level of 0.05, a desired power of 0.90, and an assumed effect size (Cohen’s d) of 0.71. The effect size of 0.71 was derived from a meta-analysis by Shin et al [25], which examined the effectiveness of simulation-based nursing education. Although this meta-analysis addressed simulation in general rather than palliative care specifically, we considered it the most relevant available estimate, as no prior studies on VCS in palliative care nursing education have reported effect sizes. We therefore adopted this conservative estimate to ensure adequate power to detect a moderate-to-large intervention effect. The sample size calculation was based on the primary outcome of palliative care knowledge, as it was considered the most sensitive to the educational intervention. Given that this is the primary outcome, no adjustment for multiplicity was applied. Taking into account a 10% attrition rate, at least 48 students per group were needed to achieve this statistical power for the study, resulting in a total target sample of 96 participants.

### 2.11. Data Collection

Seven days after their initial meeting, both groups will undergo baseline assessments of their palliative care knowledge, ability, and attitudes (pre-intervention, T0). After the intervention, post-intervention assessments (T1) will be conducted to evaluate the participants’ palliative care knowledge, ability and attitudes. In addition, students in the intervention group will be invited to complete the Student Satisfaction and Self-confidence in Learning Scale at the end of the intervention.Following the T1 assessment, the control group will receive the same VCS intervention as the intervention group.

### 2.12. Data Analysis

We will use SPSS 26.0 statistical software to analyze the data. When the data follow a normal distribution, descriptive statistics (mean and standard deviation) will be used to represent the central tendency and dispersion for continuous demographic data (e.g., age), as well as for ratings of palliative care knowledge, ability, and attitudes. For data that do not follow a normal distribution, the median and interquartile range will be utilized to depict central tendency and variability.We will use frequencies and percentages to represent categorical data, such as gender. Independent t-tests will be used to examine whether there are significant differences in the mean demographic data between the intervention and control groups. Chi-square tests will be employed to compare the distribution of sample characteristics between the two groups for homogeneity. The primary analysis to evaluate the intervention effect will be an analysis of covariance (ANCOVA) comparing post-test scores between groups, with baseline scores as a covariate. Paired t-tests will be used to assess the changes in the overall scores and scores across different dimensions of palliative care knowledge, ability, and attitudes in the students before and after the VCS education system intervention.The assumptions of normality and homogeneity of variance for parametric tests will be checked. If these assumptions are violated, appropriate non‑parametric alternatives will be considered. An intention‑to‑treat analysis will be performed. For any missing data, multiple imputation will be applied if needed. A two-tailed *P* < 0.05 is considered statistically significant.

## 3. Discussion

Palliative care is a specialized medical field within healthcare, characterized by the complexity of the situations it addresses and the irreversible nature of EOLC, which poses significant challenges in facilitating clinical practice opportunities for students in the context of EOLC. Previous studies have explored the integration of VCS into nursing education, demonstrating its effectiveness in improving students’ knowledge, ability, and self-efficacy [26–28]. However, these studies have primarily focused on general nursing skills rather than palliative care specifically, and no randomized controlled trials have been conducted in China to evaluate the impact of VCS on palliative care education for nursing students.

This RCT is designed to address this gap by rigorously testing the effectiveness of a VCS system developed specifically for palliative care education. In contrast to conventional classroom approaches, VCS recreates EOLC scenarios by dynamically depicting various stages of the terminal phase, comprehensively demonstrates care knowledge points, breaks free from the linearity of conventional classroom models, and enables students to control their learning pace independently while revisiting challenging content at their discretion.

To address this gap, we developed a VCS system tailored specifically for palliative care education and will conduct study with both an intervention and a control group.The control group will participate in conventional 90-minute classroom teaching, whereas the intervention group will receive identical classroom content, augmented by the use of the VCS system. By comparing the outcomes of these two groups, this RCT will aim to determine whether the VCS system can effectively enhance nursing students’ palliative care knowledge, ability, and attitudes.

The anticipated contributions of this study are threefold. First, it will provide the first empirical evidence from a Chinese context on the effect sizes of VCS in palliative care education. Second, it will offer insights into the feasibility of integrating VCS into an existing elective nursing course. Third, it will assess student acceptance of this innovative educational approach, which is critical for future implementation.

Several limitations of this trial should be acknowledged. First, because participants were recruited from an elective course on death and end-of-life education, students who opted into this course may already have greater interest in or more positive attitudes toward palliative care compared to the general nursing student population. This potential selection bias should be considered when interpreting the findings and may limit the generalizability of the results. Second, as a single‑institution study, the sample may not be representative of the broader nursing student population, limiting the generalizability of findings. Third, the study will only assess short‑term outcomes immediately post‑intervention, with no follow‑up to evaluate long‑term knowledge retention or behavior change in real clinical settings. Fourth, potential contamination between groups may occur if students discuss the intervention content, and all participants will be aware of their involvement in a trial, which could introduce performance bias. These limitations will be considered when interpreting the study findings.

## 4. Conclusion

This RCT will evaluate whether a VCS system developed for palliative care education can enhance nursing students’ knowledge, ability, and attitudes in this critical area. If positive effects are found, the results may inform curriculum design, support the integration of VCS into required nursing courses, and provide students with an emotionally safer environment for initial exposure to end-of-life care. The findings will ultimately provide evidence to guide nursing educators in selecting appropriate educational strategies for palliative care training.

## Supporting information

S1 File SPIRIT checklist. (PDF)

S2 File Consent form. (DOCX)

S3 File The registration study protocol. (DOCX)
