# Supplementary material for: Effectiveness of virtual clinical simulation in palliative care education for nursing students: Protocol of a randomized controlled trial
Source: PLoS One. 2026 May 28;21(5):e0349168. doi: 10.1371/journal.pone.0349168 (PMC13218514; doi:10.1371/journal.pone.0349168)
Supplement: S2 File — (DOCX) [file pone.0349168.s002.docx]

**S2 File. Consent form**

**Southwest Medical University**

**Informed Consent Form**

**Study Title**

Effectiveness of Virtual Clinical Simulation in Palliative Care Education for Nursing Students: A Randomized Controlled Trial

**Introduction**

You are invited to participate in a research study conducted by the School of Nursing at Southwest Medical University under the supervision of Professor Fu Jing.

**Purpose of the Study**

To investigate and compare the effectiveness of a virtual clinical simulation system versus traditional teaching methods in enhancing nursing students' knowledge, skills, and attitudes toward palliative care.

**Procedures**

If you agree to participate in this study, you will be randomly assigned to either the intervention group (using the virtual clinical simulation system) or the control group (receiving traditional palliative care education). The educational content for both groups is equivalent. The study will last approximately 90 minutes. You will be required to provide demographic information and complete online questionnaires and knowledge assessments at two time points: before the intervention (baseline) and immediately after the intervention. Each assessment is expected to take about 5 minutes. The total time commitment for the study is approximately 2 hours.

**Potential Risks/Stress/Pain/Discomfort/Other Factors and Their Mitigation**

The risks associated with this study are minimal. Using the virtual simulation system (if assigned to the intervention group) may cause slight eye strain or dizziness, and completing questionnaires may result in temporary fatigue. All procedures are non-invasive. You are free to take breaks during the process and may withdraw from the study at any time without penalty. If you experience any discomfort, please inform the researcher immediately.

**Potential Benefits**

By participating in this study, you will have the opportunity to learn about palliative care through innovative and structured methods. The knowledge and insights gained may benefit your future clinical practice. All educational materials and assessments related to this study will be provided free of charge.

**Participation and Withdrawal**

Your participation in this study is entirely voluntary. You have the right to withdraw at any time before or during the study without any negative impact on your academic standing or relationship with the university.

**Confidentiality**

All information collected in this study will be kept strictly confidential. Your personal identifying information (such as your name and student ID) will be replaced with a unique code. Data will be stored on a password-protected computer accessible only to the research team. The results of this study may be published in academic journals or presented at conferences, but your personal information will never be disclosed.

**Questions and Concerns**

If you have any questions or concerns about this study, please contact Professor Fu Jing at 414011449@qq.com.

**Consent Form**

Southwest Medical University

School of Nursing

Consent Form for

**Effectiveness of Virtual Clinical Simulation in Palliative Care Education for Nursing Students: A Randomized Controlled Trial**

I have read and understand the information provided about the above study. I agree to participate this study.

|  |  |  |
| --- | --- | --- |
| Name of participant | Signature of participant | Date |

|  |  |  |
| --- | --- | --- |
| Name of investigator | Signature of investigator | Date |
