# Supplementary material for: Effectiveness of virtual clinical simulation in palliative care education for nursing students: Protocol of a randomized controlled trial
Source: PLoS One. 2026 May 28;21(5):e0349168. doi: 10.1371/journal.pone.0349168 (PMC13218514; doi:10.1371/journal.pone.0349168)
Supplement: S3 File — (DOCX) [file pone.0349168.s003.docx]

**S3 File. The registration study protocol**

**Effectiveness of Virtual Clinical Simulation in Palliative Care Education for Nursing Students: Protocol of a Randomized Controlled Trial**

**Background of the study**

Cancer poses a considerable threat to global health and human life. The most recent data from the International Agency for Research on Cancer (IARC), part of the World Health Organization (WHO), indicates that in 2022, China experienced around 4.825 million new cancer cases and 2.574 million deaths attributed to cancer. These figures represent 24.2% of the global incidence and 26.4% of the global mortality associated with this disease[1]. With the rapid movement of China into an advanced stage of demographic aging, the need for palliative care within the health care system is growing. This has brought up a pressing need to enhance education and training for healthcare professionals[2,3]. Nursing students are expected to form the backbone of the future health workforce and are entrusted with the important task of providing professional end-of-life care (EOLC)[4]. By enhancing their education in palliative care, they will be able to develop a scientific perspective on death, establish positive life values, and acquire the necessary knowledge and skills to manage and adapt to death-related events, thus providing better care to patients and their families[5].Challenges of providing practical clinical education in palliative care to nursing students are distinct. Palliative care is a specialized medical field that involves proficiency in communication abilities for interactions with patients and healthcare professionals, compliance with ethics and morality as well as the complexity of end-of-life and death situations[6].Many students also become sad, afraid, and anxious upon initial exposure to EOLC, which demotivates them toward it[7]. Because these challenges in terms of emotional and education may hinder the development of their knowledge and professional ability in palliative care[8]. In view of the limitations of conventional educational approaches in meeting the dynamic needs of both education and practice in palliative care[9], there is a need to devise and implement some innovative educational models in this field.

With the help of computerized platforms, the integration of the latest developments in digital and virtual technologies introduces reconstructed scenarios observed in real life and simulates role plays.Virtual clinical simulation (VCS) marks one of the giant technological developments in the field of simulation.VCS creates immersive virtual environments for clinical training, featuring dynamic virtual patients, and allows students to engage in interactive simulations directly through their screens, offering students a simulated, hands-on experience within a controlled virtual setting[10].It is a learner-centered simulation methodology designed to develop decision-making, motor control, and communication skills, placing the individual at the core of the process[11]. VCS, as an innovative teaching approach, dynamically showcases the complex stages of EOLC, moving beyond traditional constraints of time, space, and resources to achieve educational outcomes not possible with previous models.In recent years,RCTs in countries like France, Portugal, and Singapore have implemented VCS for training nursing students. These studies have demonstrated that VCS training improves students' professional knowledge, learning satisfaction, and self-confidence, thereby enhancing their clinical judgment ability and positively impacting their clinical practice[12-14]. Integrating VCS into practical teaching substantially enhances the training abilities of students, closes the gap between theoretical learning and clinical application, and amplifies their practical and comprehensive care abilities. Nevertheless, in China, there exists a significant deficiency of interventional studies that concentrate on education in palliative care, particularly RCTs assessing the effectiveness of VCS in the training of nursing students.

**Methods**

#### Study design

This study is a prospective RCT implemented at a medical university in Southwest China. Nursing students taking the elective course "Cultural Perspectives on Death and End-of-Life Education" at the university's nursing department will be invited to participate.The study will randomly assign 96 participants into two distinct groups: a control group and an intervention group, each comprising 48 students. The control group will participate solely in conventional classroom teaching, whereas the intervention group will receive both conventional classroom teaching and enhanced learning through additional VCS.The purpose of this study is to explore whether VCS, compared to conventional teaching approaches, can enhance nursing students' knowledge, ability, and attitudes toward palliative care. The study procedure will follow the CONSORT flow diagram as shown in Fig 1.


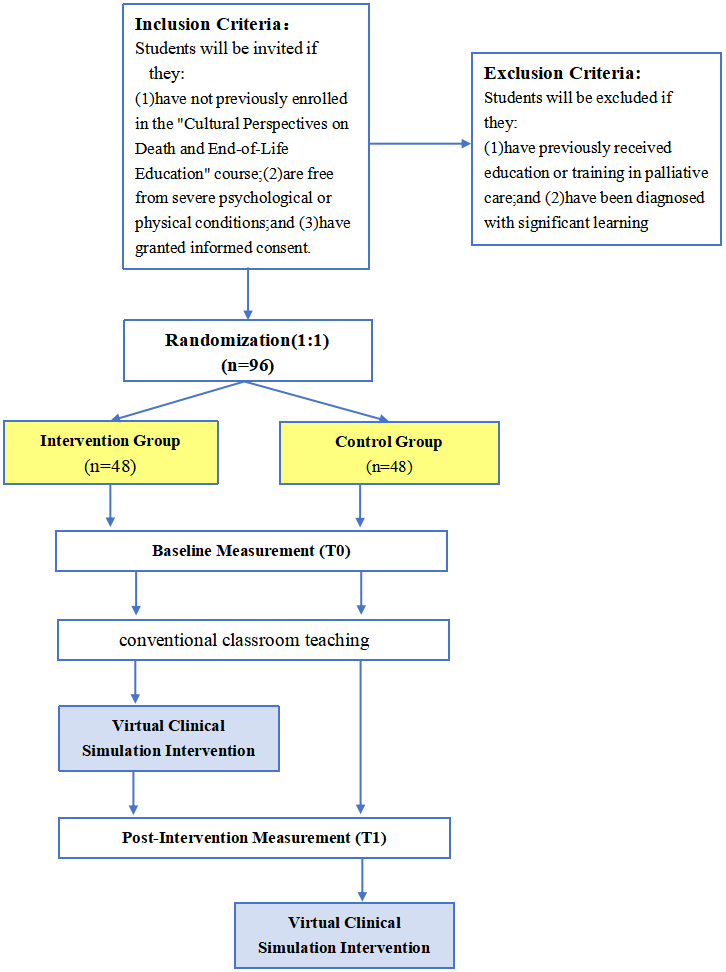


#### Fig. 1 Flowchart of the study design

#### Randomization and blinding

Every student enrolled in the course will receive an invitation via email to join as research volunteers. Those who consent will participate in an initial meeting where they will sign informed consent forms. Subsequently, these volunteers will be asked to complete a detailed questionnaire that collects their sociodemographic data and academic performance, including their average course grades.This information will then be utilized to facilitate the randomization process. To preserve confidentiality, each student will choose a unique six-digit identifier. An impartial research assistant, who will not participate in the educational activities, will utilize SPSS 26.0 statistical software to allocate students evenly into either the control or intervention group with a 1:1 ratio.Seven days after their initial meeting，and following the completion of the randomization procedure, all students who will have volunteered for the study will be invited to meeting again. This subsequent meeting will be organized to conduct a comprehensive baseline evaluation of their knowledge, ability, and attitudes concerning palliative care.This initial assessment, referred to as the pre-intervention assessment, serves to establish a benchmark against which future improvements can be measured.During this meeting, participants will be informed of their group placement through their unique identifiers and will then be directed to the appropriate classrooms for a briefing on their respective group assignments.

Due to the specific nature of the intervention, conducting a double-blind trial will be deemed unfeasible. Nonetheless, a single-blind design will be implemented by keeping the group assignments hidden from both the research assistants and the instructors.

#### Study participants

Study participants

The study will invite nursing students who have opted for the elective course "Cultural Perspectives on Death and End-of-Life Education" at a medical university in Southwest China between 1 March 2026 to 30 April 2026 to participate in this study. Written informed consent will be obtained from all participants. Students will be invited if they:(1)have not previously enrolled in the "Cultural Perspectives on Death and End-of-Life Education" course;(2)are free from severe psychological or physical conditions;and (3)have granted informed consent.Students will be excluded if they:(1)have previously received education or training in palliative care;and (2)have been diagnosed with significant learning impairments.

#### Control Group

The control group will engage in conventional classroom teaching, which will last for 90 minutes and will be conducted by a seasoned lecturer with expertise in nursing. This teaching session will mirror the key points and utilize the same case themes as those found in the VCS system.

#### Intervention Group

Participants in the intervention group will receive the same 90 minutes of classroom teaching as the control group, but they will additionally interact with a VCS system specifically developed for palliative care education by the research team.

**Intervention content**

The VCS system leverages 3D modeling technologies via Java and VRML to craft immersive environments that mimic actual hospice settings, encompassing hospital wards, medical equipment, and representations of patients, their families, and healthcare workers. Participants will assume the role of virtual nurses, engaging with non-player characters (NPCs) to adeptly navigate patient care, thereby addressing both patient and family needs.After the 90-minute conventional classroom teaching, each participant will be assigned a unique code for accessing the VCS system, which will enable research assistants to effectively track participant engagement by monitoring their login activity on the system's backend.The VCS system is meticulously organized into three comprehensive modules: the Basic Knowledge Module, the Practice Theatre Module, and the Knowledge Testing Module.In the Basic Knowledge Module(Fig.2), participants will be introduced to basic knowledge about major concepts in palliative care. Learners will acquire a solid understanding of palliative care, its core principles, essential knowledge, psychological support for the terminally ill patient, guidelines for counselling the grieving, recognition of common clinical presentation at the end of life, and updates on EOLC practices.The Practice Theatre Module focuses on the case of an 89-year-old elderly male patient with advanced lung cancer who is admitted to a palliative care ward for hospice care(Fig. 3).This module features six detailed learning scenarios: Case Introduction, Admission Care, Specialized Nursing, Spiritual Care, Social Support, and EOLC.Each scenario includes a variety of contextual educational tasks such as choices, dialogues, and reading assignments, engaging learners in decision-making processes. Learners will interact with the VCS environment by clicking on navigation icons and using menu controls on the screen, guided by an automated assistant. Additionally, the system will evaluate the "correctness" of each task choice, assign scores, and automatically log these data in the backend.Such interactions will empower virtual nurses to perform assessments, physical exams, select medical tools, execute appropriate interventions, and maintain effective communication. The overarching aim is to enhance terminally ill patients' quality of life. Furthermore, this module will seek to cultivate a compassionate attitude and holistic palliative care ability among students, thus enabling them to promptly and accurately identify and address the physiological, psychological, and emotional issues of terminally ill patients when faced with end-of-life situations. It will also promote the development of critical clinical thinking and ethical decision-making abilities. The Knowledge Testing Module will present 10 randomized questions, awarding 1 point for each correct response(Fig. 4).The system will generate experimental reports based on different scenarios, which encompass the correct task answers, the options selected by users, and their final scores.These reports will specify the steps or knowledge points that remain incomplete, and can be readily archived by users for future reference when conducting additional experiments. Furthermore, these reports will offer invaluable insights to facilitate students' reflective learning processes.


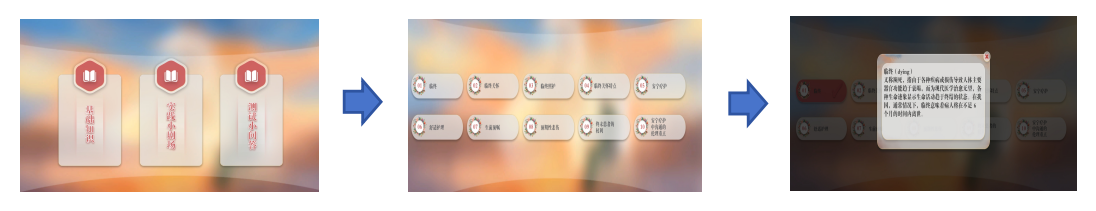


Fig. 2: The Basic Knowledge Module


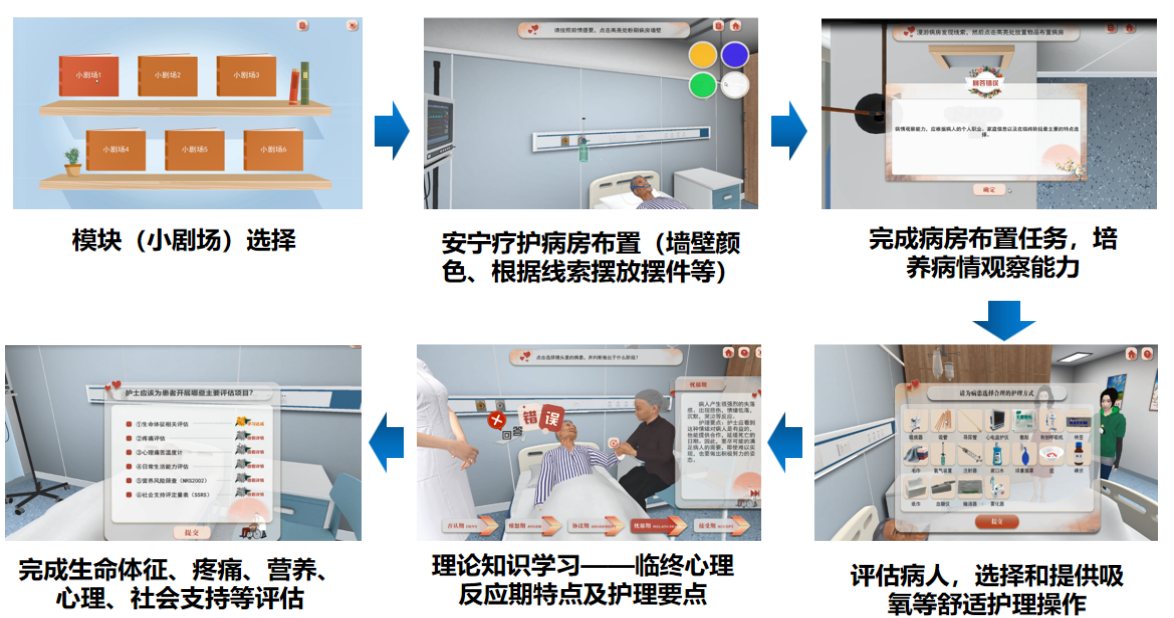


Fig. 3: The Practice Theatre Module


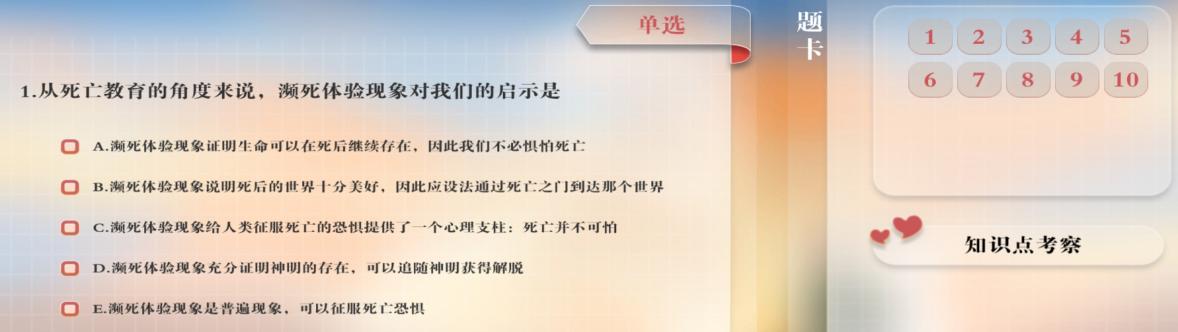


Fig. 4: The Knowledge Testing Module

**Primary Outcomes**

1.**Nurses' Palliative Care Knowledge Scale**

2.**The Undergraduate Nursing Students' Palliative Care Ability Assessment Questionnaire**

3.**The Chinese Version of the Frommelt Attitudes Toward Care of the Dying Scale Form B (FATCOD-B)**

**Secondary Outcomes**

1.Student Satisfaction and Self-confidence in Learning Scale

**Sample Size**
The determination of the necessary sample size for this investigation was conducted conducted using G*Power 3.1.9 software[15],employing a two-tailed independent t-test with an alpha level of 0.05, a power of 0.90, and an effect size of 0.71[16]. Taking into account a 10% attrition rate，at least 48 students per group were needed to achieve this statistical power for the study.

**Data Collection**
Data Collection

Seven days after their initial meeting, both groups will undergo baseline assessments of their palliative care knowledge, ability, and attitudes (pre-intervention, T0). After the intervention, post-intervention assessments (T1) will be conducted to evaluate the participants' palliative care knowledge, ability and attitudes. In addition, students in the intervention group will be invited to complete the Student Satisfaction and Self-confidence in Learning Scale at the end of the intervention.Following the T1 assessment, the control group will receive the same VCS intervention as the intervention group.

**Data Analysis**
We will use SPSS 26.0 statistical software to analyze the data. When the data follow a normal distribution, descriptive statistics (mean and standard deviation) will be used to represent the central tendency and dispersion for continuous demographic data (e.g., age), as well as for ratings of palliative care knowledge, ability, and attitudes. For data that do not follow a normal distribution, the median and interquartile range will be utilized to depict central tendency and variability.We will use frequencies and percentages to represent categorical data, such as gender. Independent t-tests will be used to examine whether there are significant differences in the mean demographic data between the intervention and control groups. Chi-square tests will be employed to compare the distribution of sample characteristics between the two groups for homogeneity. Independent t-tests will also be used to compare the levels of palliative care knowledge, ability, and attitudes between the intervention and control groups. To compare the overall scores and scores across different dimensions of palliative care knowledge, ability, and attitudes before and after the VCS education system intervention, researchers will use paired t-tests to assess the changes in these levels in the students. A two-tailed p<0.05 is considered statistically significant.

**Ethical approval and clinical trial registration**
The study has been approved by the Ethics Committee of Southwest Medical University (Approval No.:SWMUIRBTX-202501-0003). It has also been registered at the Chinese Clinical Trial Registry with the registration number ChiCTR2500096377. The Ethics Committee will review the implementation of the trial and have the authority to decide on its termination. Upon completion of the study, the research data will be uploaded to the Chinese Clinical Trials Registry.

**References**

1. Bray F, Laversanne M, Sung H, Ferlay J, Siegel RL, Soerjomataram I, et al. Global cancer statistics 2022: GLOBOCAN estimates of incidence and mortality worldwide for 36 cancers in 185 countries. CA Cancer J Clin. 2024;74(3):229-263. http://doi.org/10.3322/caac.21834 PMID:38572751

2. Rosa WE, Parekh DCA, Abedini NC, Gray TF, Huijer HA, Bhadelia A, et al. Optimizing the Global Nursing Workforce to Ensure Universal Palliative Care Access and Alleviate Serious Health-Related Suffering Worldwide. J Pain Symptom Manage. 2022;63(2):e224-e236. http://doi.org/10.1016/j.jpainsymman.2021.07.014 PMID: 34332044

3. Rosa WE, Ferrell BR, Mazanec P. Global Integration of Palliative Nursing Education to Improve Health Crisis Preparedness. J Contin Educ Nurs. 2021;52(3):130-135. http://doi.org/10.3928/00220124-20210216-07 PMID: 33631023

4. Wells G, Llewellyn C, Hiersche A, Minton O, Barclay D, Wright J. Care of the dying - medical student confidence and preparedness: mixed-methods simulation study. BMJ Support Palliat Care. 2022. http://doi.org/10.1136/spcare-2022-003698 PMID: 35850959

5. Zhang L, Huang YL, Wu XQ, Liu CY, Zhang XL, Yang XY, et al. The impact of virtual clinical simulation on nursing students' palliative care knowledge, ability, and attitudes: A mixed methods study. Nurse Educ Today. 2024;132:106037. http://doi.org/10.1016/j.nedt.2023.106037 PMID: 37976886

6. Dobrowolska B, Mazur E, Pilewska-Kozak A, Donka K, Kosicka B, Palese A. Predicted difficulties, educational needs, and interest in working in end of life care among nursing and medical students. Nurse Educ Today. 2019;83:104194. http://doi.org/10.1016/j.nedt.2019.08.012 PMID: 31493620

7. Yoong SQ, Wang W, Seah A, Kumar N, Gan J, Schmidt LT, et al. Nursing students' experiences with patient death and palliative and end-of-life care: A systematic review and meta-synthesis. Nurse Educ Pract. 2023;69:103625. http://doi.org/10.1016/j.nepr.2023.103625 PMID: 37004470

8. Tarot A, Pithon M, Ridley A, Guastella V, Plancon M, Aubry R, et al. Experiencing and enduring patient distress: the distress of palliative care patients and its emotional impact on physicians in training. BMC Med Educ. 2024;24(1):696. http://doi.org/10.1186/s12909-024-05668-9 PMID: 38926746

9. Huai P, Li Y, Wang X, Zhang L, Liu N, Yang H. The effectiveness of virtual reality technology in student nurse education: A systematic review and meta-analysis. Nurse Educ Today. 2024;138:106189. http://doi.org/10.1016/j.nedt.2024.106189 PMID: 38603830

10. Sim J, Rusli K, Seah B, Levett-Jones T, Lau Y, Liaw SY. Virtual Simulation to Enhance Clinical Reasoning in Nursing: A Systematic Review and Meta-analysis. Clin Simul Nurs. 2022;69:26-39. http://doi.org/10.1016/j.ecns.2022.05.006 PMID: 35754937

11. Liu K, Zhang W, Li W, Wang T, Zheng Y. Effectiveness of virtual reality in nursing education: a systematic review and meta-analysis. BMC Med Educ. 2023;23(1):710. http://doi.org/10.1186/s12909-023-04662-x PMID: 37770884

12. Blanie A, Amorim MA, Benhamou D. Comparative value of a simulation by gaming and a traditional teaching method to improve clinical reasoning skills necessary to detect patient deterioration: a randomized study in nursing students. BMC Med Educ. 2020;20(1):53. http://doi.org/10.1186/s12909-020-1939-6 PMID: 32075641

13. Padilha JM, Machado PP, Ribeiro A, Ramos J, Costa P. Clinical Virtual Simulation in Nursing Education: Randomized Controlled Trial. J Med Internet Res. 2019;21(3):e11529. http://doi.org/10.2196/11529 PMID: 30882355

14. Liaw SY, Chan SW, Chen FG, Hooi SC, Siau C. Comparison of virtual patient simulation with mannequin-based simulation for improving clinical performances in assessing and managing clinical deterioration: randomized controlled trial. J Med Internet Res. 2014;16(9):e214. http://doi.org/10.2196/jmir.3322 PMID: 25230684

15. Faul F, Erdfelder E, Lang AG, Buchner A. G*Power 3: a flexible statistical power analysis program for the social, behavioral, and biomedical sciences. Behav Res Methods. 2007;39(2):175-91. http://doi.org/10.3758/bf03193146 PMID: 17695343

16. Shin S, Park JH, Kim JH. Effectiveness of patient simulation in nursing education: meta-analysis. Nurse Educ Today. 2015;35(1):176-82. http://doi.org/10.1016/j.nedt.2014.09.009 PMID: 25459172
